# Bioimpedance and Dual-Energy X-ray Absorptiometry Are Not Equivalent Technologies: Comparing Fat Mass and Fat-Free Mass

**DOI:** 10.3390/ijerph192113940

**Published:** 2022-10-27

**Authors:** Sofia Lopes, Tatiana Fontes, Rejane Giacomelli Tavares, Luis Monteiro Rodrigues, Cíntia Ferreira-Pêgo

**Affiliations:** CBIOS–Universidade Lusófona’s Research Center for Biosciences and Health Technologies, Av. Campo Grande 376, 1749-024 Lisbon, Portugal

**Keywords:** bioimpedance, dual-energy X-ray absorptiometry, DXA, body fat, Fat Free-Mass

## Abstract

Bioimpedance (BIA) is the most frequently used technology for body composition assessment at a daily clinical level, mostly due to its low price and user-friendly operation. However, many doubts persist regarding its physiological meaning and applicability. The present study aimed to compare one BIA system and the Dual-Energy X-ray Absorptiometry (DXA) for the characterization of body composition in a previously selected cohort of healthy adult participants. A descriptive observational cross-sectional study included a final sample of 121 participants, 93 women and 28 men, with a mean age of 28.26 ± 9.72 years old and a mean body mass index (BMI) of 22.68 ± 3.13 kg/m^2^. Statistics involved paired *t*-tests and agreement analysis by the Bland-Altman method. BIA underestimated the percent body fat (%BF) by 5.56% and overestimated Fat-Free Mass (FFM) by 2.90 kg. A strong positive correlation between both technologies was found for FFM (r = 0.980) and the %BF (r = 0.932), but the disagreement was statistically significant (*p* < 0.001). Although DXA and BIA seem to correlate, these technologies are not congruent. Therefore, the risk of (mis)interpretation and bias is clear with BIA, potentially impacting the nutritional planning of clinical dietitians and the further results of its patients.

## 1. Introduction

Overweight and obesity have become a public health issue, especially over the last decades [1]. Defined as an excessive accumulation of body fat [2], they have been associated with cardiometabolic risk [3] and chronic processes such as Type 2 Diabetes Mellitus, Hypertension, Dyslipidemia, and Cardiovascular Diseases [1,4].

Body composition assessment is crucial to identify nutritional status and possible risks [5] and is recognized as a useful health index [6]. Anthropometric measurements have gained particular relevance as the principal technique used for overweight and obesity diagnosis [7], and for this reason, accurate and sensitive methods are needed [5,8]. The search for more accurate approaches evolved to the quantification of body mass using Bioimpedance (BIA) and Dual-Energy X-ray Absorptiometry (DXA) [9,10], among other techniques. DXA was developed to measure bone mass, which is calculated from the differential absorption of X-rays of two different energies. Because this calculation requires allowance for (and hence quantification of) overlying soft tissue, percent body fat (%BF) and fat-free mass (FFM) are also calculated for whole body scans, using instrument-specific algorithms [11]. DXA is regarded as the gold standard [12,13] for bone mass measurement [14], is one of the most accurate devices for body composition assessment, and is a fast and safe model. However, it should be kept in mind that during a measurement, the shutter opens to allow a beam of radiation to pass through the scanner table and the patient. Regarding the doses absorbed by the skin for standard size total body evaluation, the participant is exposed to a lower amount of ionizing radiation in a short timeframe, and taking into consideration the low levels of radiation absorbed by the skin [15], it seems that there is no scientific evidence indicating that the single use of DXA equipment alone is a risk factor for the individual’s health. The high (equipment) cost involved remains a major limitation to the use of DXA at a clinical level [4,6,12,14,16]. In contrast, the low cost and easy manageability of the (likewise non-invasive) BIA makes it very popular and widely used, especially with large population samples [6,12,13,17], and mainly by dietitians and personal trainers. The consistency of its physical principle is still controversial as most BIA techniques lack further validity and demonstration of accuracy [18]. There are different forms of BIA devices—single and multi-frequency—which can achieve different levels of accuracy [11]. Moreover, BIA does not provide a direct measurement but rather an estimation of body composition based on predictions obtained from specific populations [13]. High errors are expected when the sample is different from the pattern [8,13,19], and thus BIA validity has yet to be demonstrated [20]. It is important to note that the system is designed to provide an estimation by measuring the resistance of an undetectable electric current passing through the body [8,13], being lower in tissues with high water contents, such as FFM [8]. For this reason, the accuracy of the results has many influencing factors, such as hydration, external temperature, caffeine consumption, exercise, and others [21]. In addition, BIA reads as if the body were a homogeneous cylinder [17], with the limitations magnified in the analysis of obese subjects [22].

The objective of the present analysis was to compare the %BF and FFM assessed through the BIA and DXA devices and analyze their agreement in an adult population.

## 2. Materials and Methods

### 2.1. Study Design and Population

The present study consisted of a cross-sectional design with a sample of 121 participants with a mean age of 28.26 ± 9.72 years, presenting a mean height of 1.67 ± 0.08 m, mean weight of 63.22 ± 11.41 kg, and consequently a mean BMI of 22.68 ± 3.13 kg/m^2^, with a classification of normal weight according to WHO guidelines [23]. Recruitment took place from March 2021 to December 2021. Non-inclusion criteria included taking any regular medication or food supplementation that was incompatible with evaluation, being under 18 years old, and being pregnant (or potentially pregnant) or breastfeeding. Volunteers made a one-time visit to the laboratory, the evaluations were performed using the same equipment as with the study, and all the participants were dressed in lightweight clothes with no metal accessories. All measurements were collected according to the same chronology: (1) sociodemographic survey; (2) body weight; (3) BIA; and (4) DXA measurements. The study was conducted according to the principles of the Helsinki Declaration [24], and all the participants signed the written informed consent included in the study. The study protocol was approved by the School of Sciences and Health Technologies Ethics Committee (CE.ECTS/P05-21).

### 2.2. Sociodemographic Data

A survey was specially designed to address the general characteristics of the subjects, such as sex, age, area of residence, academic degree, educational qualifications, area of study, and monthly family income, among others. Other lifestyle questions were also assessed, for example, smoking status, supplementation, medication use, and quality and hours of sleep.

### 2.3. Anthropometry and Body Composition

During the assessment, subjects were instructed to fast for at least 12 h, including abstaining from the consumption of alcoholic beverages, coffee, or caffeine, and with no practice of physical activity. Height was collected from each subject’s Portuguese nationality card and body weight was evaluated using a BIA (*Tanita BC 545N^®^)*. With these data, BMI was calculated using the Quetelet formula [body weight (kg)/[height (m) × eight (m)] [25]. In all BIA measurements (single frequency device), the subjects were barefoot without socks. Participants placed their feet and hands on the electrodes, and were asked to remain still until the signal finished the measurement. This type of BIA equipment was selected considering its wide use in some clinical practice and gyms in Portugal. The DXA (Lunar Prodigy Advance—General Electric Healthcare^®^; Chicago, IL, USA) was used to measure bone mass, body fat, lean mass, tissue mass, fat-free mass, total mass, and visceral and subcutaneous adipose tissue. Before each whole-body scan, the DXA was calibrated according to the manufacturer’s instructions via a standard calibration block. Participants removed shoes, socks, and all jewelry. Wearing undergarments or close-fitting clothing with no metallic pieces, participants were instructed to lie supine on the scanning bed with hands by their sides, not touching the body. During all body scans, participants were asked to remain motionless and Velcro straps were situated around the ankles and knees. All participants on the DXA scanning bed were positioned on the scanning bed by the same trained researcher. Scans lasted approximately 5 to 10 min. The researcher analyzed each scan to adjust software-determined regions of interest before producing the whole-body report. The measurements of BIA and DXA occurred under the same conditions of temperature, clothing, fasting, etc., reducing the bias between individuals.

### 2.4. Statistical Analysis

Nominal variables are expressed as percentages and frequencies, and continuous variables as mean and standard deviation (SD). Percentage differences were obtained using the percentage variation formula [(Vf-Vi)/(Vi) × 100]. The significant differences and correlations between the FFM and %BF values provided by DXA and BIA were analyzed with the paired *t*-test. Bland-Altman [26] was performed to analyze the agreement between the devices for these variables, with the limits calculated through ± 1.96 SD. Quintiles for BIA and DXA values of FFM and %BF were calculated. The degree of gross misclassification in the differences between the two methods was evaluated using contingency tables. The proportions of correctly categorized subjects are in the same quintiles. All statistical tests were two-tailed, and the significance level was set at *p* < 0.05. All analyses were performed using the SPSS software version 27.0 (SPSS Inc., Chicago, IL, USA).

## 3. Results

The demographic characteristics according to sex are described in Table 1. Statistically significant differences were observed regarding height, weight, BMI, and exercise practice, with males showing higher values for all these variables.

Table 2 describes the differences in FFM and %BF data provided by both instruments (BIA and DXA). The percentage differences in the general population showed a greater difference between the measurements of %BF compared to FFM (22.16% versus 6.92%, respectively). Regarding sex, there was a greater percentage of difference in males than in females for %BF (38.46% versus 19.49%, respectively) not observed for FFM (6.45% versus 7.13%, respectively).

Table 3 shows the data correlation from both technologies.

**Figure 1:**
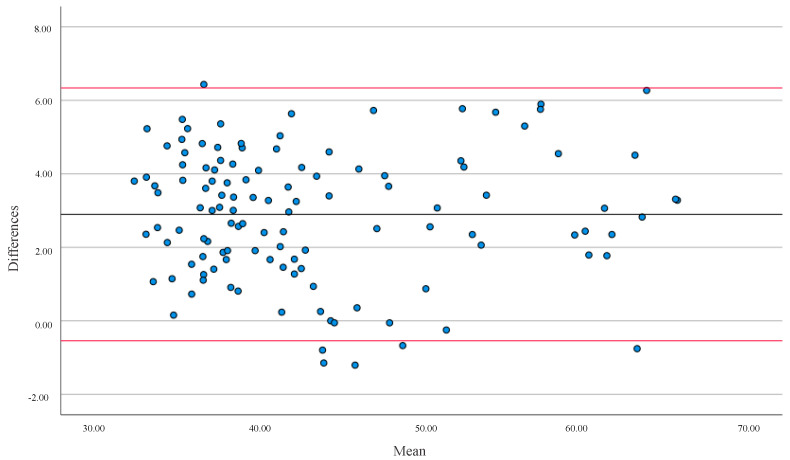
Bland-Altman analysis evaluated the differences in Fat-Free Mass (FFM) in kg between the two types of equipment (BIA and DXA). The solid line represents the Mean (2.90) and the red lines represent ± 1.96 SD for all populations. Upper limit = 6.34 SD; lower limit = −0.54 SD.

**Figure 2:**
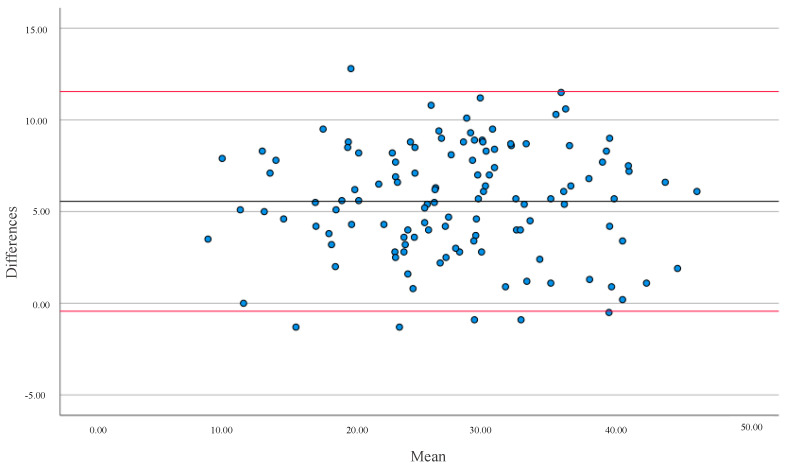
Bland-Altman analysis evaluated the differences in Percentage Body Fat (%BF) between the two equipment (BIA and DXA) versus the Averages between the values. The solid line represents the Mean (5.56) and the red lines represent ± 1.96 SD for all populations. Upper limit = 11.55 SD; lower limit = −0.43 SD.

In both cases, the correlation between methods was positive, with a high correlation in FFM and %BF for the general population (Figure 1 and Figure 2). The mean differences were 5.56% for %BF and 2.90 kg for FFM. In both cases, there was no agreement between both devices (*p* < 0.001).

Table 4 verified the percentage of misclassification bias (overestimation or underestimation of BIA relative to DXA) using a contingency table. The between-methods analysis classified 66.10% of the individuals in the same quintile concerning %BF and 70.30% concerning FFM.

## 4. Discussion

The purpose of the present study was to compare DXA and BIA evaluations on %BF and FFM in a healthy population with a wide age range of adults. Our results have shown that both instruments are strongly correlated regarding FFM and %BF, with statistically significant differences between instruments involving wide limits of agreement, in line with recent reports [16,27]. By checking the misclassification bias through the contingency table, we observed quite positive values, where 66.10% and 70.30% of participants were in the same quintile of %BF and FFM percentages, respectively. Further analysis of these 121 individuals has shown that approximately 41 and 36 are not in the same %BF and FFM quintile percentages, respectively. This BIA misclassification might negatively influence the interpretation and impact of dietary planning [21]. BIA seemed to consistently underestimate %BF by 5.56% and overestimate FFM by 2.90 kg. These differences are aligned with previous studies [7,10,28]. In 2012, Leahy et al. [28] reported an underestimate of 2.1% for %BF and a overestimate ≈ of 1 kg for muscle mass. In our study, men have shown a higher underestimation of %BF compared to women (5.85% vs. 5.47%) in opposition to previously published works [1,28]. Likely related to the sample’s low homogeneity, where men presented a higher BMI compared to women, this has been associated with differences among instruments [4,6,13,16,17].

There is a growing interest in the use of BIA to assess body composition in multiple settings, including research. The arguments are obvious when using BIA, ranging from portability, ease of use, cost, and lack of radiation, to practical operation. However, BIA results are critically affected by multiple assumptions based on predictive equations that condition the instrument estimations and their applicability [6,29], and the physiological meaning of the results remains in doubt. From another perspective, credible comparative studies between BIA and DXA are still insufficient and often conflicting [28,29,30,31]. Major criticisms affecting the validity of these studies range from different populations even if healthy (size, age, sex, race, body mass) to the use of different BIA (manufactured instruments), thus not comparable equations [9]. However, if it could be determined that BIA measures have a systematic bias compared to reference methods, it could be that they are accounted for, in practice. In addition, for conclusive statements to be made about the accuracy and usefulness of BIA, studies would need to assess its reliability/repeatability and would need to compare it with other criterion measures, such as MRI or CT, or direct measures such as total body water or total body counting and neuron activation. In conclusion, although BIA technology has greatly improved during the last years, has become evident that further studies are needed with regard to this medical technology to demonstrate its effectiveness compared to other commonly used methods [32]. Some limitations should be assumed, such as the sample population’s reduced dimension and inhomogeneity, mainly in the sex distribution; also the cross-sectional design did not allow prospective body composition comparisons. However, despite these limitations, this study adds to the knowledge that BIA and DXA are dissimilar equipment for %BF and FFM assessment.

## 5. Conclusions

Our study consistently confirms the presence of relevant estimation errors with BIA compared to DXA in the presence of a strong positive correlation between the two instruments. The Bland-Altman analysis also has shown that these techniques were discordant (*p* < 0.001), confirming that BIA and DXA are dissimilar types of equipment for %BF and FFM assessment. Further studies and expanded experimental design are needed to better understand BIA’s physiological significance and its application to body composition studies. Concluding, the risk of (mis)interpretation and bias that can occur with this type of BIA equipment means that it is not the perfect option for body composition assessment, potentially impacting the nutritional planning of clinical dietitians and further results of its patients. It is important to alert professionals to the need to implement the best clinical procedures in body composition assessment.

## Figures and Tables

**Table 1:** General characteristics of the study population categorized by sex.

	All Population (*n* = 121)	Male (*n* = 28)	Female (*n* = 93)	*p*-Value ^a^
**Age, years**	28.26 (9.72)	30.32 (8.98)	27.65 (9.18)	0.177
**Height, m**	1.67 (0.08)	1.77 (0.06)	1.63 (0.06)	<0.001
**Weight, kg**	63.22 (11.41)	72.42 (7.89)	60.46 (10.87)	<0.001
**BMI, kg/m^2^**	22.68 (3.13)	23.16 (2.29)	22.54 (3.34)	0.356
**Monthly Family Income, % (n)**				
Without answer	11.60 (14)	10.70 (3)	11.80 (11)	0.762
<1000€	11.60 (14)	7.10 (2)	12.90 (12)	
1000€–3000€	63.60 (77)	71.40 (20)	61.30 (57)	
>3000€	13.20 (16)	10.70 (3)	14.00 (13)	
**Residence Area, % (n)**				
Urban	81.80 (99)	85.70 (24)	80.60 (75)	0.542
Rural	18.20 (22)	14.30 (4)	19.40 (18)	
**Dietary Pattern, % (n)**				
Vegetarian/Vegan	37.20 (45)	42.90 (12)	35.50 (33)	0.479
Omnivore	62.80 (76)	57.10 (16)	64.50 (60)	
**Academic course, % (n)**				
None	2.50 (3)	3.60 (1)	2.20 (2)	0.346
Nutrition	51.20 (62)	39.30 (11)	54.80 (51)	
Other	46.30 (56)	57.10 (16)	43.00 (40)	
**Physical Activity, practice % (n)**	59.50 (72)	78.60 (22)	53,80 (50)	0.019
**Smoker, % (n)**	14.90 (18)	17.90 (5)	14.00 (13)	0.613

Data expressed as mean (standard deviation) or percentage (*n*). Abbreviations: BMI, Body Mass Index. ^a^ *p*-values for group comparisons were tested by Student’s *t*-test or Pearson’s χ^2^ as appropriate.

**Table 2:** Differences in fat-free mass and fat mass between the two methods studied (BIA and DXA).

	Fat-Free Mass, Kg		Body Fat, %	
	DXA	BIA	DXA	BIA
**General Population (n = 121)**	41.92 (8.80)	44.82 (8.81)	30.65 (8.31)	25.09 (8.32)
Differences	2.90		5.56	
Differences, %	6.92		22.16	
**Female (n = 93)**	38.01 (4.82)	40.72 (4.20)	33.54 (6.51)	28.07 (6.56)
Differences	2.71		5.47	
Differences, %	7.13		19.49	
**Male (n = 28)**	54.89 (6.18)	58.43 (5.92)	21.06 (6.19)	15.21 (5.41)
Differences	3.54		5.85	
Differences, %	6.45		38.46	

Data expressed as mean (standard deviation). Percentual differences were calculated using the formulas FFM = ([(BIA − DXA)/(DXA)] × 100), and %BF = ([(DXA-BIA)/(BIA)] * 100).

**Table 3:** Correlation between fat-free mass and body fat was analyzed by the two methods studied (BIA and DXA).

	Correlation	*p*-Value ^a^
**DXA Fat-Free Mass, kg**	0.980	<0.001
**BIA Fat-Free Mass, kg**		
**DXA Body Fat, %**	0.932	<0.001
**BIA Body Fat, %**		

Data expressed as mean (standard deviation). Percentual differences were calculated using the formulas FFM = ([(BIA − DXA)/(DXA)] × 100), and %BF = ([(DXA − BIA)/(BIA)] × 100). ^a^ *p*-values for group comparisons were tested by Student’s *t*-test or Pearson’s χ^2^ as appropriate.

**Table 4:** Contingency tables for the percentage of misclassification between the quintiles of the two equipment (BIA and DXA) regarding body fat and fat-free mass.

**Quintiles of DXA Body Fat values (%)**	**Quintiles of BIA Body Fat Values (%)**					
		**Q1**	**Q2**	**Q3**	**Q4**	**Q5**
	**Q1**	18.2	0.8	0.8	0.0	0.0
	**Q2**	1.7	13.2	5.0	0.0	0.0
	**Q3**	0.0	5.8	8.3	5.8	0.8
	**Q4**	0.0	0.0	7.4	9.9	2.5
	**Q5**	0.0	0.0	0.0	3.3	16.5
**Quintiles of DXA Fat-Free Mass values (%)**	**Quintiles of BIA Fat-Free Mass values (%)**					
		**Q1**	**Q2**	**Q3**	**Q4**	**Q5**
	**Q1**	14.9	5.0	0.0	0.0	0.0
	**Q2**	6.6	9.1	4.1	0.0	0.0
	**Q3**	0.0	4.1	13.2	3.3	0.0
	**Q4**	0.0	0.0	3.3	14.9	1.7
	**Q5**	0.0	0.0	0.0	1.7	18.2

## Data Availability

Not applicable.

## References

[1] Silveira E.A., Barbosa L.S., Rodrigues A.P.S., Noll M., De Oliveira C. (2020). Body fat percentage assessment by skinfold equation, bioimpedance and densitometry in older adults. Arch. Public Health.

[2] Tsatsoulis A., Paschou S.A. (2020). Metabolically Healthy Obesity: Criteria, Epidemiology, Controversies, and Consequences. Curr. Obes. Rep..

[3] Krachler B., Völgyi E., Savonen K., Tylavsky F.A., Alén M., Cheng S. (2013). BMI and an anthropometry-based estimate of fat mass percentage are both valid discriminators of cardiometabolic risk: A comparison with DXA and bioimpedance. J. Obes..

[4] Day K., Kwok A., Evans A., Mata F., Verdejo-Garcia A., Hart K., Ward L.C., Truby H. (2018). Comparison of a bioelectrical impedance device against the reference method dual energy X-ray absorptiometry and anthropometry for the evaluation of body composition in adults. Nutrients.

[5] Benito P.J., Gómez-Candela C., Cabañas M.D., Szendrei B., Castro E.A. (2019). Comparison between different methods for measuring body fat after a weight loss program. Rev. Bras. Med. Esporte.

[6] Achamrah N., Colange G., Delay J., Rimbert A., Folope V., Petit A., Grigioni S., Déchelotte P., Coëffier M. (2018). Comparison of body composition assessment by DXA and BIA according to the body mass index: A retrospective study on 3655 measures. PLoS ONE.

[7] Del Velazquez-Alva M.C., Irigoyen-Camacho M.E., Huerta-Huerta R., Delgadillo-Velazquez J. (2014). Comparación de la absorciometría de rayos x de energia dual y dos analizadores de impedancia bioeléctrica para medir el porcentaje de grasa corporal y el índice de masa libre de grasa en un grupo de mujeres jóvenes mexicanas. Nutr. Hosp..

[8] Wingo B.C., Barry V.G., Ellis A.C., Gower B.A. (2018). Comparison of segmental body composition estimated by bioelectrical impedance analysis and dual-energy X-ray absorptiometry. Clin. Nutr. ESPEN.

[9] Ramírez-Vélez R., Tordecilla-Sanders A., Correa-Bautista J.E., González-Ruíz K., González-Jiménez E., Triana-Reina H.R., García-Hermoso A., Schmidt-RioValle J. (2018). Validation of multi-frequency bioelectrical impedance analysis versus dual-energy X-ray absorptiometry to measure body fat percentage in overweight/obese Colombian adults. Am. J. Hum. Biol..

[10] Meier N.F., Bai Y., Wang C., Lee D.C. (2020). Validation of a multielectrode bioelectrical impedance analyzer with a dual-energy x-ray absorptiometer for the assessment of body composition in older adults. J. Aging Phys. Act..

[11] Wells J.C.K., Fewtrell M.S. (2006). Measuring body composition. Arch. Dis. Child..

[12] Antonio J., Kenyon M., Ellerbroek A., Carson C., Burgess V., Tyler-Palmer D., Mike J., Roberts J., Angeli G., Peacock C. (2019). Comparison of dual-energy x-ray absorptiometry (DXA) versus a multi-frequency bioelectrical impedance (InBody 770) device for body composition assessment after a 4-week hypoenergetic diet. J. Funct. Morphol. Kinesiol..

[13] Rassel C.R., Bewski N.A., O’Loughlin E.K., Wright A., Scheel D.P., Puig L., Kakinami L. (2019). Validity of electrical impedance myography to estimate percent body fat: Comparison to bio-electrical impedance and dual-energy X-ray absorptiometry. J. Sports Med. Phys. Fitness.

[14] Lee S.Y., Ahn S., Kim Y.J., Ji M.J., Kim K.M., Choi S.H., Jang H.C., Lim S. (2018). Comparison between dual-energy x-ray absorptiometry and bioelectrical impedance analyses for accuracy in measuring whole body muscle mass and appendicular skeletal muscle mass. Nutrients.

[15] Williams K.M., Darukhanavala A., Hicks R., Kelly A. (2022). An update on methods for assessing bone quality and health in Cystic fibrosis. J. Clin. Transl. Endocrinol..

[16] Wang Z.H., Yang Z.P., Wang X.J., Dong Y.H., Ma J. (2018). Comparative Analysis of the Multi-Frequency Bio-impedance and Dual-energy X-ray Absorptiometry on Body Composition in Obese Subjects. Biomed. Environ. Sci..

[17] Liao Y.S., Li H.C., Lu H.K., Lai C.L., Wang Y.S., Hsieh K.C. (2020). Comparison of bioelectrical impedance analysis and dual energy X-ray absorptiometry for total and segmental bone mineral content with a three-compartment model. Int. J. Environ. Res. Public Health.

[18] Davydov D.M., Boev A., Gorbunov S. (2021). Making the choice between bioelectrical impedance measures for body hydration status assessment. Sci. Rep..

[19] Xu L., Cheng X., Wang J., Cao Q., Sato T., Wang M., Zhao X., Liang W. (2011). Comparisons of Body-Composition Prediction Accuracy: A Study of 2 Bioelectric Impedance Consumer Devices in Healthy Chinese Persons Using DXA and MRI as Criteria Methods. J. Clin. Densitom..

[20] Talma H., Chinapaw M.J.M., Bakker B., Hirasing R.A., Terwee C.B., Altenburg T.M. (2013). Bioelectrical impedance analysis to estimate body composition in children and adolescents: A systematic review and evidence appraisal of validity, responsiveness, reliability and measurement error. Obes. Rev..

[21] Carvalho P., Editora P. (2021). Os Novos Mitos Que Comemos.

[22] Ballesteros-Pomar M.D., Calleja-Fernández A., Diez-Rodríguez R., Vidal-Casariego A., Blanco-Suárez M.D., Cano-Rodríguez I. (2012). Comparación de las diferentes medidas de la composición corporal en pacientes con obesidad grave en un contexto clínico. Nutr. Hosp..

[23] (2004). World Health Organization Body mass index. Kans. Nurse.

[24] (2014). World Medical Association World Medical Association Declaration of Helsinki: Ethical principles for medical research involving human subjects. J. Am. Coll. Dent..

[25] Garrow J.S., Webster J. (1985). Quetelet’s index (W/H2) as a measure of fatness. Int. J. Obes..

[26] Martin Bland J., Altman D.G. (1986). Statistical methods for assessing agreement between two methods of clinical measurement. Lancet.

[27] Yang S.W., Kim T.H., Choi H.M. (2018). The reproducibility and validity verification for body composition measuring devices using bioelectrical impedance analysis in Korean adults. J. Exerc. Rehabil..

[28] Leahy S., O’Neill C., Sohun R., Jakeman P. (2012). A comparison of dual energy X-ray absorptiometry and bioelectrical impedance analysis to measure total and segmental body composition in healthy young adults. Eur. J. Appl. Physiol..

[29] Ellegård L., Bertz F., Winkvist A., Bosaeus I., Brekke H.K. (2016). Body composition in overweight and obese women postpartum: Bioimpedance methods validated by dual energy X-ray absorptiometry and doubly labeled water. Eur. J. Clin. Nutr..

[30] Thomson R., Brinkworth G.D., Buckley J.D., Noakes M., Clifton P.M. (2007). Good agreement between bioelectrical impedance and dual-energy X-ray absorptiometry for estimating changes in body composition during weight loss in overweight young women. Clin. Nutr..

[31] Gába A., Kapuš O., Cuberek R., Botek M. (2015). Comparison of multi- and single-frequency bioelectrical impedance analysis with dual-energy X-ray absorptiometry for assessment of body composition in post-menopausal women: Effects of body mass index and accelerometer-determined physical activity. J. Hum. Nutr. Diet..

[32] Lopes A.A., Albuquerque L., Fontes M., Rego D., Bandeira F. (2022). Body Composition in Acromegaly According to Disease Activity—Performance of Dual X-Ray Absorptiometry and Multifrequency Bioelectrical Impedance Analysis. Front. Endocrinol..

